# The Combined Effect of Intravenous and Topical Tranexamic Acid in Liposuction: A Randomized Double-Blinded Controlled Trial

**DOI:** 10.1093/asjof/ojab002

**Published:** 2021-01-12

**Authors:** Nicolas M Abboud, Ayush K Kapila, Sofie Abboud, Elie Yaacoub, Marwan H Abboud

**Affiliations:** 1 Plastic and Reconstructive Surgery Department, Centre Hospitalier Universitaire de Tivoli, La Louvière, Université Libre de Bruxelles (U.L.B.), Brussels, Belgium; 2 Department of Plastic and Reconstructive Surgery at the University Hospital Brussels (UZ Brussel), Brussels, Belgium; 3 Université Libre de Bruxelles (U.L.B.), Brussels, Belgium

## Abstract

**Background:**

Tranexamic acid (TXA) use in surgical procedures due to its hemostatic effects has been gaining an increased interest. In plastic surgery, the effects of TXA have been studied intravenously (IV), and there have been some reports regarding local use.

**Objectives:**

A comparative study examining the combined effect of IV and local TXA was conducted.

**Methods:**

A randomized double-blinded controlled trial was performed for patients undergoing breast reduction treatment with liposuction and resection following the power-assisted liposuction mammaplasty (PALM) technique. All patients received 5 mL IV of 0.5 g/5 mL TXA on induction. Before installation, one researcher prepared two solutions of 1 L normal saline: one with 5 mL of 0.5 g/5 mL TXA associated with epinephrine 1:100,000 and the other with only epinephrine 1:100,000. These were randomly infiltrated in either the left or right breast. Clinical dermal bleeding was assessed for both breasts after deepithelialization. The lipoaspirate from these breasts was then compared with each other. A postoperative evaluation at 24 hours was performed to compare the ecchymosis rate.

**Results:**

Ratios of decanted volume to total lipoaspirate was measured in bottles and compared between breasts. There was a statistical difference (*P* = 0.0002) in the ratio of decanted to lipoaspirated volume when comparing the control group (ratio: 0.21) with the treatment group (0.13). Video analysis revealed decreased dermal bleeding in the TXA group and postoperative evaluation less ecchymosis.

**Conclusions:**

The combined use of IV and local TXA can help reducing blood loss in liposuction as measured by decantation in separate drain bottles and as assessed clinically preoperatively and postoperatively.

**Level of Evidence: 2:**

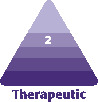

Tranexamic acid (TXA), well known for its antifibrinolytic properties,^1^ has been of increasing interest in plastic surgery procedures since the last decade. In other specialties, such as cardiac surgery, orthopedic surgery, and gynecology, TXA has been used to good effect, both intravenously (IV) and topically.^2-6^ Rohrich and Cho^2^ published a review on TXA use in 2018 and found increasing potential in plastic surgery. Murphy et al^3^ performed a meta-analysis in 2016 and noted that TXA was able to reduce blood loss and transfusion requirements in craniomaxillofacial and plastic surgery. Furthermore, Brown et al^7^ reported the benefits of the combined use of local and IV TXA in rhinoplasty, clearing the operative field, decreasing the preoperative bleeding, and reducing postoperative edema and ecchymosis. Similar findings were observed by Nayak and Linkov^8^ while using a 0.75% of TXA infiltration solution in facelifts and a 1% of TXA solution in rhinoplasty.

In different surgical specialties, TXA has been used both IV and local. Early in vitro work on TXA showed the ideal dose to be 10 mg/kg.^3,9^ In our practice, we have been using TXA both IV and local in combination. We hypothesize that the combined effect benefits efficacy while avoiding potential systemic side effects of over-administration in IV. To analyze the combined effect of topical TXA in infiltration before liposuction with IV TXA given at induction, we performed a double-blinded randomized controlled trial at our center.

## METHODS

### Patient Selection

A prospective randomized double-blinded study was performed from August 2019 till February 2020. In total, 36 patients of breast reduction by liposuction and resection (PALM)^10,11^ were included in this study. During the procedure, one breast would receive local infiltration with TXA and one without. This is randomized and explained further in the surgical technique. Both patient and surgeon were blinded to which of the two infiltrates is used per breast.

Exclusion criteria for the study were: use of antiplatelets or anticoagulants, previous thromboembolism, history of cardiovascular incidents or surgery, previous evidence of genetic predisposition to thromboembolism (such as Factor V Leiden disease, and thrombophilia), or bleeding (such as hemophilia and von Willebrand factor disease).

All of the patients received detailed information of the surgical technique and of the risks and benefits of various surgical options. Written consent forms were provided by these patients. The study is in accordance with the Declaration of Helsinki guidelines. Because all patients underwent surgical procedures in a private practice, approval from an institutional review board or ethics committee was not obtained.

### Surgical Technique

The first step is patient preparation. Preoperatively, a thorough clinical history is obtained, and patients with the above exclusion criteria are excluded from the study. Patients who smoke are told to quit 4 weeks before the procedure, as is routine for all patients operated by the senior author.

On the day of the operation, patients are marked. Once in the operating theater, our anesthetic colleagues give 5 mL of 0.5 g/5 mL of IV Exacyl (TXA; Sanofi-Aventis France, Gentilly, France) at induction. Hereafter, the patient is installed. Before infiltration, the primary researcher would have already prepared the infiltrating solution accordingly in a different room. One solution would contain 5 mL of Exacyl (Sanofi-Aventis France, Gentilly, France) 0.5 g/5 mL associated with epinephrine 1:100,000 per liter of normal saline. Another solution would contain epinephrine 1:100,000 per liter of normal saline. Both of them are randomly connected to a power-assisted liposuction system (Lipomatic Eva SP, Euromi SA, Verviers, Belgium), which is initially used for infiltration on the machine’s infiltration mode.

The operator initially infiltrates one of the breasts as per his choosing with either the bag with TXA or the bag without TXA depending on which bag was randomly connected to run first by the researcher. Hereafter, the second bag is connected. Before infiltration of the solution in the second bag, the infiltrate is purged to ensure that there is no mixing of the two solutions. Then, the second solution is infiltrated by the same operator. The researcher keeps a track on which bag is used for each breast. After 20 minutes, a deepithelization is performed according to the power-assisted liposuction mammaplasty (PALM) technique,^10^ and sharp blades are replaced when deepithelializing the second breast. Dermal bleeding from both breasts is assessed, and videos are taken by the researcher. Liposuction of the breast that was initially infiltrated is then initiated; this is for the breast of either the treatment or the control group depending on which solution was initially randomly used for the breast that was first infiltrated. The lipoaspirate is collected through a closed system in a drain bottle. Once the operator decides to lipoaspirate the contralateral breast, the drain bottle is changed by the assistant. After lipoaspiration of both breasts, the drain bottles are placed next to each other and the aspirate is allowed to decant. After 60 minutes of decantation, the researcher takes a note of the ratio of decantated plasma to the entire lipoaspirate volume.

Postoperative evaluation at 24 hours is performed by researchers for every patient, and a comparison is made of ecchymosis and hematoma rate in both breasts.

## RESULTS

### Patient Characteristics

In total, 36 patients with an average age of 39 (range: 19-66) and average body mass index (BMI) of 29 (range: 24-35) were included in the study. All of the patients (72 breasts) had breast reduction by liposuction and resection following the PALM technique;^4^ 8 of the 36 patients were smokers (22%). There were no postoperative incidences of thrombosis or hematoma requiring revisional surgery (Table 1).

**Table 1.** Patient Characteristics

Characteristic	Result
Number of patients	36 patients
Number of breasts	72 breasts
Average age	39 years (range: 19-66)
Average body mass index	29 kg/m^2^ (range: 24-35)
Smokers	8 patients (22%)

### Procedure Characteristics

Infiltration time per breast by using the power-assisted liposuction machine was on average 2 minutes (range: 1-3 minutes). Per breast, an average of 370 mL was infiltrated (range: 240-500 mL). Deepithelialization was performed on average 22 minutes after induction (range: 16-35 minutes). On average, 690 cc (range: 350-1200 cc) of lipoaspirate was suctioned per breast; this was comparable in both the control and treatment groups of breasts. Procedure time was on average 87 minutes (range: 63-113 minutes; Table 2).

**Table 2.** Procedure Characteristics

Characteristic	Average
Infiltration time	2 min (range: 1-3 min)
Infiltration volume	370 cc (range: 240-500 cc)
Time before deepithelialization	22 min (range: 16-35 cc)
Lipoaspirated volume	690 cc (range: 350-1200 cc)
Procedure time	87 min (range 63-113 min)

### Ratio Analysis

**Figure 1.**
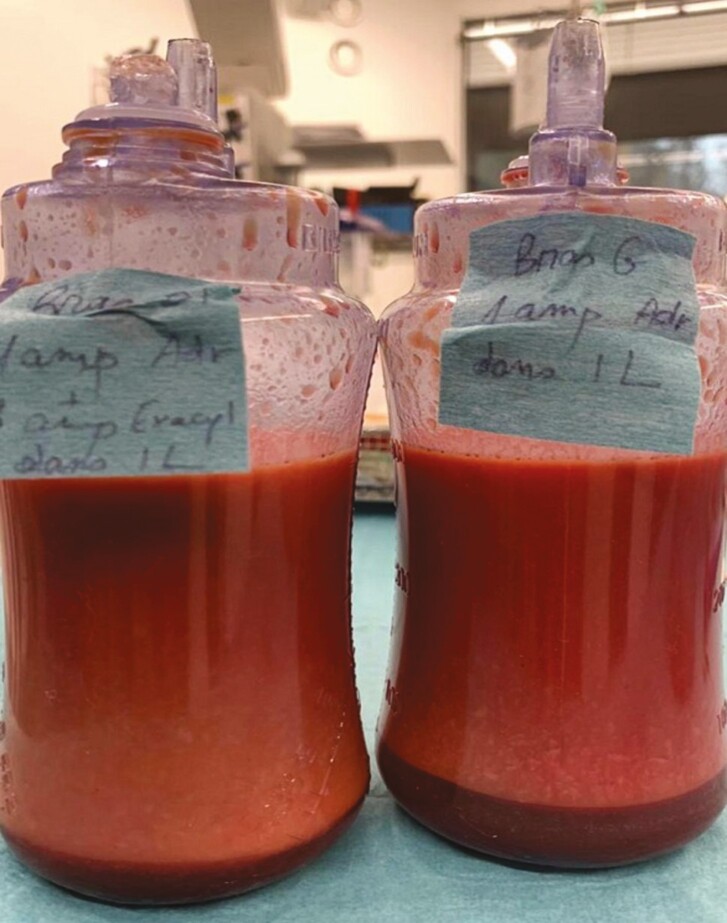
Example of decantation in drain bottles for a 46-year-old female patient, patient A. On the left bottle, the lipoaspirate is from the right breast, where a combination of tranexamic acid (TXA) 0.5 g (in 5 mL) associated with epinephrine 1:100,000 per liter of normal saline was used for infiltration. On the right side, the lipoaspirate in the bottle is from the left breast where a solution containing epinephrine 1:100,000 per liter of normal saline was used. The patient had 0.5 g (in 5 mL) of TXA on induction intravenously. We note that the ratio of decanted volume to total lipoaspirate is 20:300 on the right and 50:300 on the left breast.

**Figure 2.**
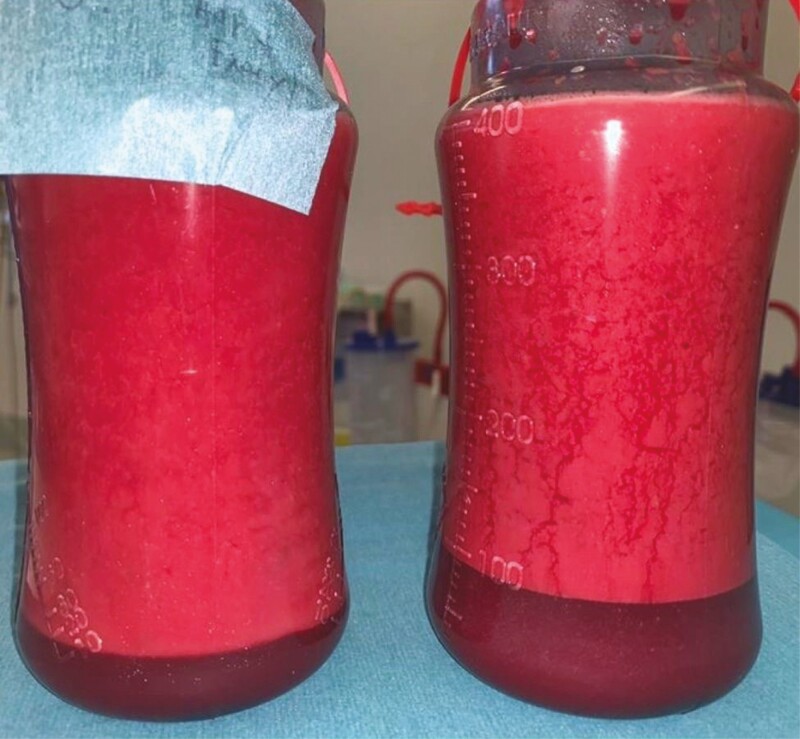
Example of decantation in drain bottles for a 40-year-old female patient, patient B. On the left bottle, the lipoaspirate is from the right breast where a combination of tranexamic acid (TXA) 0.5 g (in 5 mL) associated with epinephrine 1:100,000 per liter of normal saline was used for infiltration. The right-side bottle represents lipoaspirate from the left breast where a solution containing epinephrine 1:100,000 per liter of normal saline was used. The patient had 0.5 g (in 5 mL) of TXA on induction intravenously. We note that the ratio of decanted volume to total lipoaspirate is 50:400 on the right and 90:400 on the left breast.

The ratio of decanted volume of blood to total lipoaspirated volume in a drain bottle was analyzed. For the control group (epinephrine alone), the average ratio was 0.21 (range: 0.09-0.36). For the treatment group (epinephrine and TXA), the average ratio was 0.13 (range: 0.04-0.20). There was a 38% reduction in the ratio in the treatment group when compared with control. Unpaired *t*-test analysis revealed a *P*-value of 0.0002 showing statistical significance. Examples are shown in Figures 1, 2 and Table 3.

**Table 3.** Ratio Analysis

	Control group	Treatment group
Ratio	0.21 (range: 0.09-0.36)	0.13 (range: 0.04-0.20)
*P*-value	0.0002	

### Video Analysis

**Figure 3.**
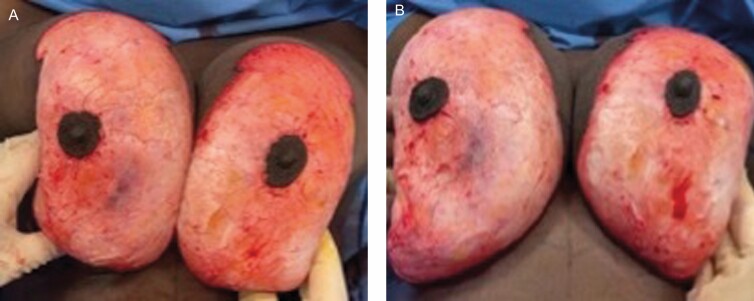
Assessment of dermal bleeding on a 46-year-old female patient, patient A. The operator was blinded to the infiltrate solution used for each breast; this was 1 liter normal saline with either one with 5 mL of 0.5 g/5 mL tranexamic acid (TXA) associated with epinephrine 1:100,000 or with only epinephrine 1:100,000. In this patient, the right breast was infiltrated with 300 mL of a solution of 5 mL of 0.5 g/5 mL TXA associated with epinephrine 1:100,000 in 1 L of normal saline. The left breast was infiltrated with 300 mL of a solution of epinephrine 1:100,000 in 1 L of normal saline. (A) A first photograph is taken at the start of the video just as the two large gauzes are taken off. (B) A second photograph is taken at the end of the video reflecting increasing amount of dermal bleeding on the right when compared with the left.

**Figure 4.**
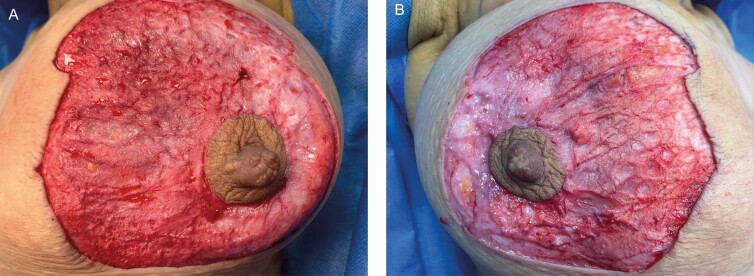
Assessment of dermal bleeding on a 40-year-old female patient, patient B. The operator was blinded to the infiltrate solution used for each breast; this was 1 liter normal saline with either one with 5 mL of 0.5 g/5 mL tranexamic acid (TXA) associated with epinephrine 1:100,000 or with only epinephrine 1:100,000. In this patient, (A) the right breast was infiltrated with 300 mL of a solution of 5 mL of 0.5 g/5 mL TXA associated with epinephrine 1:100,000 in 1 L of normal saline. (B) The left breast was infiltrated with 300 mL of a solution of epinephrine 1:100,000 in 1 L of normal saline. Both pictures were taken at the end of the video reflecting increasing amount of dermal bleeding on the right when compared with the left.

Anonymized video analysis by two researchers who were not in the operating theater showed reduced dermal bleeding after deepithelialization in PALM breast reduction with scar in the TXA group. This was noted in 31 of the 36 patients, and in 5 patients this was similar. This was done by putting two large gauzes one for each breast to dry the dermis. The gauzes were taken off and a video was taken to assess bleeding during 90 seconds between the two breasts. The operator was blinded to which of the two solutions (with or without TXA) was infiltrated into the breasts. Examples of photos taken at the start and/or at the end of the video (Videos 1-3) can be seen on Figures 3, 4.

### Photograph Analysis

Two researchers who were not in the operating theater performed an anonymized photograph analysis at 24 hours postoperatively for completion of the study. They estimated an increased ecchymosis rate in the treatment group for all of the patients.

## DISCUSSION

The interest in TXA has significantly increased for reducing blood loss and the need of transfusion in many surgical specialties; however, its effect in plastic surgery remains underexplored, except in the field of craniofacial surgery.^2-6^ Bearing this evidence in mind, along with our results, the use of a combination of local TXA infiltration with preinduction IV TXA has real potential. The senior author has been utilizing this solution for more than 5 years in breast surgery, body contouring, brachioplasty, face and neck lifts, as well as rhinoplasty. It is, therefore, that a formal analysis of the data has been performed to demonstrate the impact in a simple and clear manner.

Usually given IV, with the usual dosage ranging from 0.5 to 10 g, early in vitro work on TXA suggested that for TXA to inhibit fibrinolysis, one has to ensure a concentration of 10 µg per milliliter in vitro.^9^ This would translate in 10 mg/kg, in order to block 80% of the plasminogen activation. Nevertheless, other authors would maintain a dosage superior to the therapeutic threshold.^9^ More recently, Cansancao et al^12^ published a prospective, double-blind, nonrandomized study to analyze the efficacy of IV TXA in reducing perioperative blood loss during liposuction. The treatment group received 10 mg/kg of TXA IV preoperatively and postoperatively and compared this with a placebo in the control group. The authors found significantly decreased volume of blood loss for every liter of lipoaspirate in the TXA group when compared with the control group.^12^

Besides IV use, local TXA infiltration remains of interest as well. Recent evidence suggests that the topical use of TXA may be comparable than IV use in terms of hemostasis, thereby clearing the surgical field and facilitating the surgery. This might also diminish the administered dose of epinephrine, not only reducing consequently its adverse effects, such as tachycardia and hypertension,^13-15^ but also limiting the IV dose of TXA in high-risk patients, lowering the risk of thromboembolic events.^16^ Zilinsky et al^17^ explored the use of topical TXA in dermatologic surgery. In their study, the treatment group received lidocaine 2% diluted 1:1 with TXA 100 mg/1 mL preoperatively compared with the control group that received lidocaine 2% diluted 1:1 with normal saline. The authors assessed bleeding on Telfa pads and noted decreased blood loss in patients receiving anticoagulants.^17^ The same applies to breast reduction surgery, where the only randomized clinical trial using a topical solution of 2.5% TXA showed a reduced bleeding of 39%.^18^

We believe that a combination of both IV TXA and local infiltration of TXA can provide an adequate hemostatic effect, while preventing systemic complications as shown by Ausen et al.^19^ In our study, 5 mL of 0.5 g/5 mL of TXA was injected IV for all of our patients regardless of the weight, combined to a local administration in an infiltration solution containing 5 mL of 0.5 g/5 mL TXA with epinephrine 1:100,000 per liter of normal saline. The mean infiltration volume was 370 cc (range: 240-500 cc), which represents 185 mg of TXA received locally in average. We noted reduced dermal bleeding clinically as well as a 38% decreased “decanted volume-to-total lipoaspirated volume” ratio. Moreover, less ecchymoses were observed at 24 hours postoperatively, even though this photographic analysis is more an estimation to supplement the findings of the study. A more objective measurement is underway to quantify ecchymosis using a digitalized platform.

Since each of our patients received TXA IV, we cannot exclude the fact that it might have influenced the local action of TXA in the study group, resulting in less ecchymoses, blood loss, and dermal bleeding. Local TXA has recently shown its benefits in reducing bleeding, swelling, and bruising in face, breast, and body contouring procedures,^2,7,8,12,20-22^ but further studies should be conducted in order to analyze its local use without being combined to an IV administration, in breast and body contouring procedures.

In our study, hematocrit and other blood values from the lipoaspirate were not measured as it was not technically feasible to measure these values from the lipoaspirate in our laboratory. Furthermore, we wanted to reduce any possible bias between patients; hence, both the control arm and the treatment arm were performed on the same patient. As such, measuring blood values would not help in comparing the treatment to the control arm in the study.

Nonetheless, we do recognize that there are some limitations to our study. First of all, the possibility of the varying duration and aggression by which liposuction is performed from one breast to another. Nonetheless, 72 breasts were analyzed, which we believe is a high enough sample to offset this potential bias. Furthermore, the assessment of clinical dermal bleeding may be impacted by the depth of deepithelialization. This was performed 20 minutes after infiltration and would enable a better understanding of the local effect of TXA, before fat extraction. To offset the limitation that the depth of deepithelialization may differ between breasts during the procedure, this was always done by the same operator and according to the same protocol. Another potential limitation is that ecchymosis is not only influenced by liposuction but also by resection. Nonetheless, for all 3 of these points, we feel that the blinded nature of the study along with the number of cases can help in offsetting these. During the course of the study, we ensured only one experienced operator performed the entire procedure, and we maintained a strictly double-blinded protocol. As such, over the course of 72 breasts, we hope to balance as much as possible the natural surgical variability that one operator may have.

## CONCLUSIONS

Our double-blinded randomized controlled trial demonstrates that using a combination of preinduction IV dose of 0.5 g in 5 mL TXA with local TXA infiltration of 0.5 g in 5 mL TXA associated with epinephrine 1:100,000 per liter of normal saline can help reducing blood loss in liposuction. The combination of IV and local TXA allows optimal efficacy in our experience, while minimizing systemic complications.
